# Mechanics and Failure Mechanisms of Rigid–Flexible 3D-Printed FRP Miura-*Ori* Structures

**DOI:** 10.3390/ma19112293

**Published:** 2026-05-28

**Authors:** Zhiyu Qiao, Jitao Liu, Minghao Fan, Haifei Zhuang, Xiangyu Wang, Jiaying Xu, Peng Wang, Shaofeng Qin, Teng Wang, Weiwen Li

**Affiliations:** 1 Guangdong Provincial Key Laboratory of Durability for Marine Civil Engineering, College of Civil and Transportation Engineering, Shenzhen University & China National Key Laboratory of Green and Long-Life Road Engineering in Extreme Environment (Shenzhen), Shenzhen 518060, China; 2 CSCEC Road and Bridge Group Co., Ltd., Shijiazhuang 050011, China; 3 Department of Civil Engineering, The Hong Kong University of Science and Technology, Hong Kong, China; 4 Department of Civil Engineering, The University of Hong Kong, Hong Kong, China

**Keywords:** 3D printing, dual-material hinge, FRP Miura-*ori* structure, failure mechanism

## Abstract

**Highlights:**

- A 5.0 mm interlaced hinge effectively suppresses premature interfacial debonding in dual-material 3D-printed FRP Miura-*ori* structure.
- The optimized geometry shifts the dominant failure mechanism from weak interfacial separation to ductile fracture within the TPU elastomer.
- Hinge optimization improves system toughness, increasing fracture elongation from 100.8% to 342.4% without sacrificing peak tensile strength.
- Dual-material Miura-*ori* architecture exhibits 28.5% higher compressive capacity and 133.8% greater fracture elongation than a single-material printed structure.

**Abstract:**

The integration of multi-material 3D printing with origami engineering offers a promising avenue for deployable structures, but weak interfacial bonding between rigid and flexible phases remains a key limitation. This study first proposed four distinct hinge designs (enclosed, interlaced, inserted, and interlocked) for Miura-*ori* architectures, and subsequently investigated their mechanical behaviors with further elucidation of stress-transfer efficiency and interfacial failure modes under static tensile or compressive loading. Research outcomes identified the 5.0 mm interlaced hinge as the optimal interface design, improving stress distribution at the rigid–flexible interface and suppressing premature debonding. Notably, the dominant failure mode shifted from interfacial separation to ductile fracture within a TPU elastomer. Further research proves that increasing the embedment depth of the interlaced hinge from 1.0 mm to 5.0 mm can significantly increase fracture elongation from 100.8% to 342.4% while maintaining a stable peak tensile strength of approximately 12.5 MPa. At the structural scale, dual-material printed Miura-*ori* architecture exhibits better mechanical performance than single-material printed spatial counterparts (5163 N vs. 4019 N in compressive capacity, 18.7 mm vs. 8.0 mm in fracture elongation). These findings provide valuable insights into high-performance deployable structure design based on multi-material additive manufacturing.

## 1. Introduction

The global shift toward sustainable and resilient engineering systems requires new types of structural systems across all fields (not limited to civil) [1,2]. These systems must be capable of bearing weight and be lightweight, easy to deploy, and adaptable to different project needs. Even though conventional structure systems (especially for solid steel and concrete structures) perform effectively under stable conditions, their application in rapidly changing environments remains limited. Their considerable self-weight poses challenges for transportation and on-site installation, whereas their fixed structural configurations constrain adaptability and limit the potential for subsequent modification. In particular, emergency situations (e.g., natural disasters) demand rapid response, efficient use of space, and material reusability. Conventional structure systems usually struggle to satisfy these requirements. Therefore, the development of new structural systems and more adaptable manufacturing methods has become an urgent priority in modern engineering [3].

Deployable structures have attracted increasing attention in engineering because they can transition between compact and expanded configurations while maintaining structural functionality. Among these systems, origami-inspired architectures provide an efficient geometric strategy for transforming flat sheets into three-dimensional load-bearing forms through predefined crease patterns [1,2]. Their ability to achieve large shape change, high packing efficiency, and rapid deployment has led to extensive investigations in aerospace, robotics, biomedical devices, and lightweight structural design [3,4,5,6].

Within this broader context, the Miura-*ori* pattern is particularly attractive because it combines a simple tessellated geometry with well-defined kinematic behavior, making it a representative platform for studying the mechanical performance of foldable structures [7,8]. Miura-ori allows materials to achieve very high levels of compression, indicating that structures can be transported in tightly folded forms. Workers can then deploy them on-site within hours. This process saves space during transport and supports faster construction in urgent situations [8,9,10,11,12].

Translating origami concepts into engineering requires high-performance materials. Conventional origami structures usually use pure polymers [4], facing durability issues in aggressive environments. In contrast, continuous fiber-reinforced polymer (FRP), especially carbon-based FRP (CFRP), provides high specific strength and excellent durability, satisfying the engineering application demand [13,14,15,16]. Even so, conventional manufacturing methods (e.g., handmade and pultrusion) create a major bottleneck for FRP origami. Manual folding and adhesive assembly usually produce structures with low precision and unreliable joints, which may become critical weak points. As a result, they limit the use of more complex geometries. Multi-material fused deposition modeling (FDM) offers a promising solution. This method allows precise placement of rigid materials, (e.g., polylactic acid, PLA) and elastomeric materials (e.g., thermoplastic polyurethane, TPU) within a single fabrication process [17]. In Miura-ori structures, this accurate material distribution helps the facets remain flat and load-bearing. Moreover, this approach preserves sufficient crease flexibility for repeated folding while eliminating the need for post-fabrication assembly. It also reduces common failure modes, particularly interfacial delamination, and enables precise control of folding behavior and mechanical performance through local adjustment of crease thickness, deposition paths, and material gradients. Through mold-free co-extrusion of dissimilar materials, FDM enables the seamless combination of rigid FRP panels and flexible polymer hinges, which can eliminate weak adhesive joints and substantially improve overall structural integrity [18].

Despite this strong potential, the 3D printing of dual-material FRP Miura-ori structures is still at an early stage. Several basic mechanical and manufacturing challenges continue to limit its development. One major problem is the weak interfacial bonding between different polymer matrices during co-extrusion [19]. Weak interfacial bonding often leads to premature delamination and defect propagation. The other critical issue concerns the flexible hinge, which governs both folding behavior and stress transfer, yet still lacks well-defined and systematic design criteria. It should be noted that the global failure behavior of 3D-printed FRP origami structures is strongly influenced by process-induced anisotropy and defect sensitivity [20]. These inherent uncertainties continue to impede their broader engineering implementation. Most importantly, the micromechanical behavior of 3D-printed flexible hinges has not yet been comprehensively characterized, and the optimal geometric configurations, together with their associated ultimate failure mechanisms, remain insufficiently understood.

This paper presents a systematic investigation of 3D-printed dual-material FRP Miura-ori structures, with particular attention to structural design, micromechanical behavior, and macroscopic mechanical performance. To address the central challenge associated with the interface between flexible hinges and rigid panels, four hinge configurations were designed and experimentally examined. The effects of embedment depth and hinge thickness on load-carrying capacity and deformability were quantitatively assessed to determine an effective structural configuration under static conditions. The underlying micromechanical failure modes are further analyzed, with emphasis placed on distinguishing interfacial debonding. These insights are then extended to the structural level of single- and dual-material Miura-ori structures. Their compressive mechanical response in terms of both load-bearing capacity and fracture elongation is evaluated. By elucidating the global failure behavior and interfacial stress-transfer mechanisms, this study addresses critical technical challenges in multi-material additive manufacturing. The findings provide a theoretical foundation and a scalable design strategy for the development of intelligent, reconfigurable, and resilient composite infrastructure.

## 2. Experimental Program

### 2.1. Materials

The fabrication of 3D-printed flexural hinges requires a multi-material design strategy [21]. This strategy must balance two conflicting needs. The structure must be stiff enough to carry loads. It must also be flexible enough to undergo repeated folding. Conventional single-material fused deposition modeling (FDM) hinges often use rigid polymers such as polylactic acid (PLA) or acrylonitrile butadiene styrene (ABS) [22]. These hinges are prone to brittle fracture and anisotropic delamination under cyclic loading. In contrast, purely elastomeric materials such as thermoplastic polyurethane (TPU) do not provide enough stiffness to maintain geometric stability under service loads [23]. To solve this problem, we separate the functions of load-bearing rigidity and hinge flexibility within the structure. We achieve this separation through a mechanically interlocked dual-material design. This approach avoids the need for impractical layer-by-layer adhesive bonding or traditional mechanical fasteners. Instead, it relies on the integrated coupling of a high-strain thermoplastic elastomer with a high-stiffness composite matrix [24].

To provide the hinge with sufficient flexibility, energy dissipation, and fatigue resistance, we selected thermoplastic polyurethane (TPU) as the elastomeric material. TPU possesses these properties because of its block copolymer microstructure. This structure contains alternating soft and hard segments. The soft segments, which are usually polyester or polyether, provide extensibility. The hard segments, which are formed from diisocyanates, act as physical cross-links. In this study, we used TPU-95A (Shore hardness 95A). We chose this semi-flexible grade because it offers a good balance between large-deformation foldability and printability in fused deposition modeling (FDM) [25]. Softer elastomers often create processing problems during printing. By contrast, TPU-95A supports more stable extrusion, reduces the risk of nozzle clogging, and provides enough recovery force for hinge action. At the same time, it also offers good abrasion resistance and chemical stability [23].

For the rigid load-bearing panels, we used a composite material made of polyamide 6 reinforced with 20 wt% short carbon fibers (PA6-CF). This material combines the toughness of the PA6 matrix with the high stiffness of carbon fibers. As a result, it shows much better tensile strength, flexural modulus, and thermal stability than unreinforced polyamide [26]. Carbon fiber reinforcement is also important for additive manufacturing. The fibers help limit volumetric shrinkage during printing. They also reduce thermal warping during the layer-by-layer deposition process. Because of this, the printed parts achieve better dimensional accuracy and greater geometric stability [27]. The material also produces a surface quality that is suitable for structural components in deployed origami systems.

### 2.2. Fused Deposition Modeling

**Figure 1:**
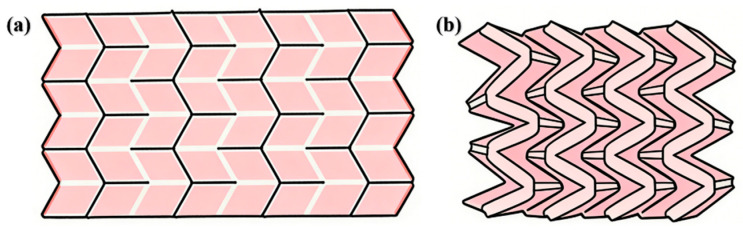
Miura-ori structure: (**a**) spreading, (**b**) folding [28].

Fused deposition modeling (FDM) has become one of the most important additive manufacturing methods for building integrated kinematic structures, including origami-inspired deployable systems [28] (Figure 1). This method is especially useful because it supports multi-extrusion printing. As a result, it can continuously place different polymers in specific locations during a single process. This capability is essential for creating single-material structures with spatially controlled stiffness and flexibility. Compared with other additive manufacturing methods, dual-material FDM offers a clear advantage. It allows rigid load-bearing panels and flexible elastomeric hinges to be printed together in one step. This process removes the need for secondary assembly [29].

The dual-extrusion FDM process uses computer-controlled deposition of thermoplastic filaments. The printer follows toolpaths that are generated from sliced three-dimensional CAD models. During printing, the material is heated above its melting temperature and deposited layer by layer. The deposited material then cools rapidly and solidifies. This process promotes bonding between adjacent layers. For this study, the dual-extrusion platform was especially important [30]. This system allows independent control of two thermal processing paths. As a result, it can continuously print and bond materials with different structures and chemical properties at the same interface.

**Figure 2:**
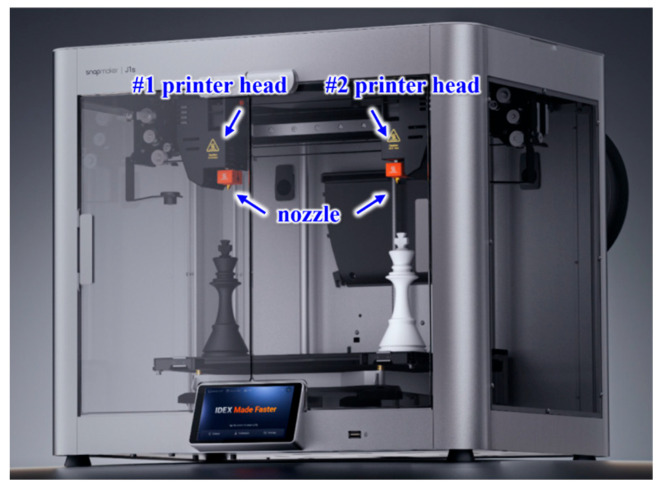
Snapmaker J1S printer.

In this work, we designed the multi-material origami geometries in SOLIDWORKS and sliced the models in Ultimaker Cura v5.12. We fabricated all specimens using an independent dual-extruder printer (Snapmaker J1s in Figure 2, Snapmaker Technologies Co., Ltd., Shenzhen, China). We set different processing parameters for each material to improve flow behavior during printing and strengthen interfacial adhesion after deposition. We extruded the elastomeric TPU-95A at 220 °C with a printing speed of 100 mm/s. We printed the composite PA6-CF at a higher temperature of 275 °C and a lower speed of 80 mm/s. These settings help ensure sufficient matrix melting and supported carbon fiber alignment. Both print heads used a 0.4 mm nozzle. We kept the layer height at 0.2 mm and the extrusion width at 0.4 mm for all samples. We also used a unidirectional raster pattern with a 0° orientation. This setting aligns the deposited filaments with the main loading direction. As a result, it improves the tensile performance and longitudinal stiffness of the printed structures.

### 2.3. Flexible Hinge Design

The structural performance of 3D-printed dual-material origami depends heavily on the effectiveness of the flexible hinges. These hinges must allow folding while still transferring stress between rigid panels. However, the large difference in modulus between stiff PA6-CF panels and soft TPU fold lines often creates a serious problem. The weak natural adhesion between these two materials can lead to early interfacial debonding under loading. To address this basic manufacturing challenge, we designed four different flexible hinge architectures. Our goal was to improve the balance between folding compliance and overall load-bearing capacity. The four designs are the enclosed, interlaced, inserted, and interlocked configurations. Each design uses a different geometric and processing strategy to control stress transfer between the two materials and reduce interfacial failure. These strategies range from full elastomeric encapsulation to precise mechanical interpenetration guided by the printing toolpath. A parametric study was conducted for each hinge design by varying the embedment length ($L_{e}$) from 1 mm to 5 mm. Four replicate specimens were evaluated for each test condition. Thus, a total of 64 specimens were prepared in this study.

**Figure 3:**
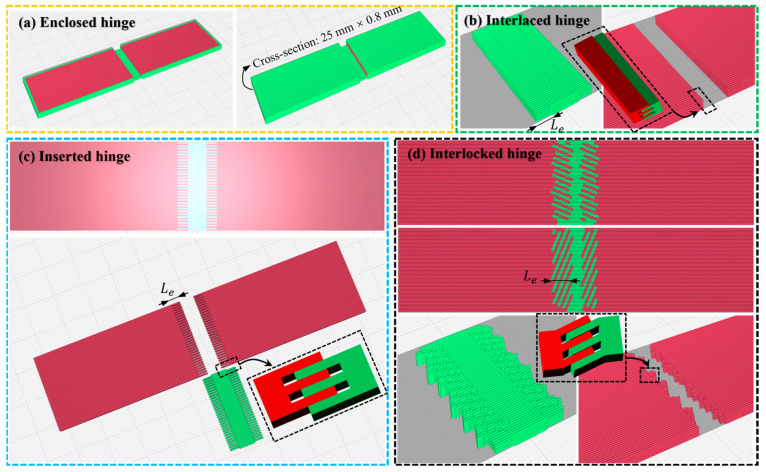
Schematic representation of diverse flexible hinge geometries: (**a**) enclosed hinge, (**b**) interlace hinge, (**c**) inserted hinge, (**d**) interlocked hinge.

*Enclosed hinge*. The enclosed hinge architecture fully surrounds a rigid composite core with a continuous elastomeric matrix (Figure 3a). In this design, the outer flexible layer acts as the main pivot during folding. This configuration avoids localized interfacial stress concentrations at the ends of the flexural joint. As a result, both the folding behavior and the ultimate tensile capacity depend mainly on the continuous elastomeric phase. By completely enclosing the rigid panels, this design also avoids the weak interfacial bonding that usually occurs between the TPU and PA6-CF regions.

*Interlaced hinge*. In contrast, the interlaced hinge uses a compliant TPU core that interpenetrates with rigid PA6-CF boundary panels (Figure 3b). We fabricated this bi-material interface through alternating cross-layer deposition during FDM. This arrangement improves stress transfer across the interface. It does so through both local thermoplastic fusion and mesoscale mechanical interlocking. As a result, the design provides the flexibility needed for deployable origami structures while maintaining overall structural integrity. It also keeps out-of-plane bending resistance low, which helps the hinge fold more easily.

*Inserted hinge*. For the inserted hinge (Figure 3c), we designed parallel wall-like micro-ribs in the overlap region between the elastomeric joint and the rigid PA6-CF panel. This configuration offers an important advantage over more complex computationally generated interlocking meshes. Its geometry works in direct harmony with the FDM process. We aligned the macroscopic teeth parallel to the main loading direction. This alignment allows the deposition path to remain longitudinal. It also keeps the carbon fiber reinforcement in the PA6-CF aligned in the same direction. As a result, the unidirectional raster pattern reduces the intra-layer voids and filament bonding defects that often appear in multidirectional printing paths.

*Interlocked hinge*. The interlocked hinge (Figure 3d) uses matching geometric features, including interpenetrating protrusions and recesses, to form a closed mechanical connection. This design supports in situ print-in-place fabrication. As a result, it removes the need for post-processing or manual assembly. During printing, synchronized extrusion paths in the upper and lower layers create an integrated interlocking structure after consolidation. To reduce the interfacial weakness that often appears in multi-material printing, we further refined the joint design. We achieved this improvement by introducing ultrafine mechanical interlocking through local toolpath control during the slicing process.

### 2.4. Folding Miura Structure

**Figure 4:**
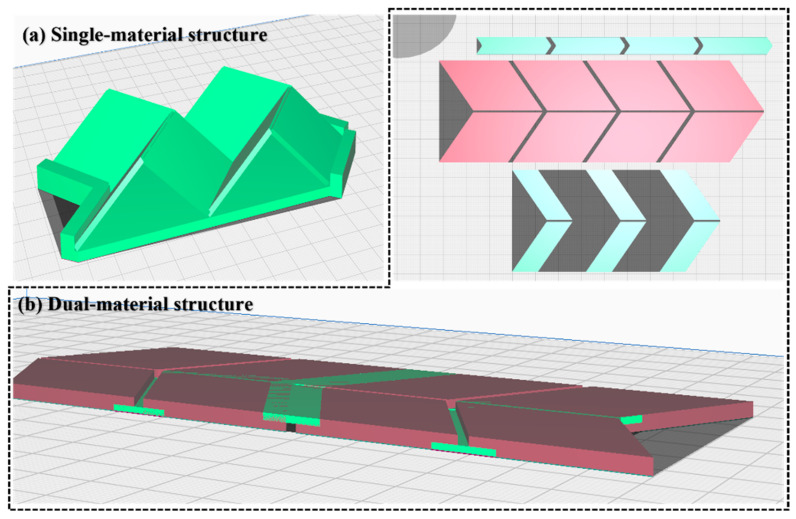
Schematic representation of diverse folding Miura structures: (**a**) single-material, (**b**) dual-material.

The single-material Miura-ori unit is computationally modeled comprising rhombic panels (30 mm side length, 60° acute angle, 1.8 mm thickness). The folded configuration is established by defining a 90° dihedral angle between adjacent longitudinal panels and anchored to a 3 mm-thick conformal basal plate prior to computational slicing (Figure 4a). Conversely, the dual-material structures incorporate interlaced elastomeric hinges (0.8 mm thickness) spanning a 2 mm inter-panel gap. To optimize the kinematic compliance and global foldability of the architecture, the hinge placement is geometrically bifurcated: valley creases are integrated at the superior panel surfaces, while mountain creases are positioned at the inferior surfaces (Figure 4b). Four replicate specimens are tested for both the single-material and dual-material FRP Miura-ori structures.

### 2.5. Testing Procedure

**Figure 5:**
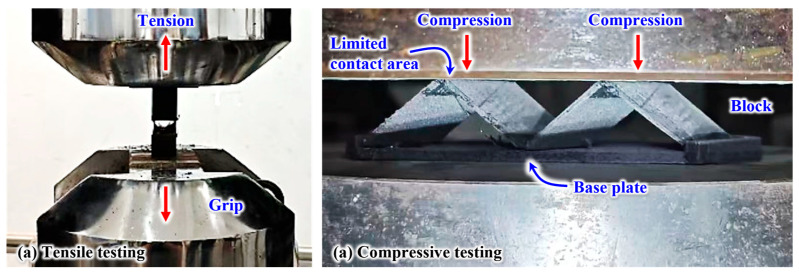
Setups for (**a**) tensile and (**b**) compressive testing.

*Tensile characterization of dual-material hinges*. To evaluate interfacial stress transfer and ultimate mechanical capacity, we performed uniaxial tensile tests using an MTS universal testing machine (MTS Industrial Systems (China) Co., Ltd., Shanghai, China) (Figure 5a). We fixed the rigid PA6-CF panels in aligned grips to avoid eccentric loading. We then applied a constant displacement rate of 8 mm/min. Stress–strain profiles were recorded until structural failure to precisely differentiate between premature PA6-CF/TPU interfacial debonding and ultimate hyperelastic rupture within the TPU matrix.

*Compressive testing of Miura-ori structures*. To characterize macroscopic load-bearing capacity and folding kinematics, 3D-printed Miura-ori structures underwent uniaxial compression on the MTS system equipped with parallel rigid steel platens (Figure 5b). Blocks were positioned on a basal plate to offer lateral constraints, restraining free lateral movement and permitting natural kinematic expansion during compression at a constant rate of 8 mm/min. Global compressive responses were monitored to capture the three distinct phases of structural evolution: initial elastic resistance, the progressive energy-absorbing folding plateau, and ultimate macroscopic densification.

## 3. Results and Discussion

### 3.1. Tensile Behavior of Dual-Material Hinges

The macroscopic resilience of single-material 3D-printed origami is intrinsically bottlenecked by the mechanical reliability of its dual-material flexible hinges. To determine the optimal geometric configuration for mitigating interfacial stress concentrations and preventing premature delamination, we systematically characterized the uniaxial tensile behavior of the four engineered hinge architectures.

**Figure 6:**
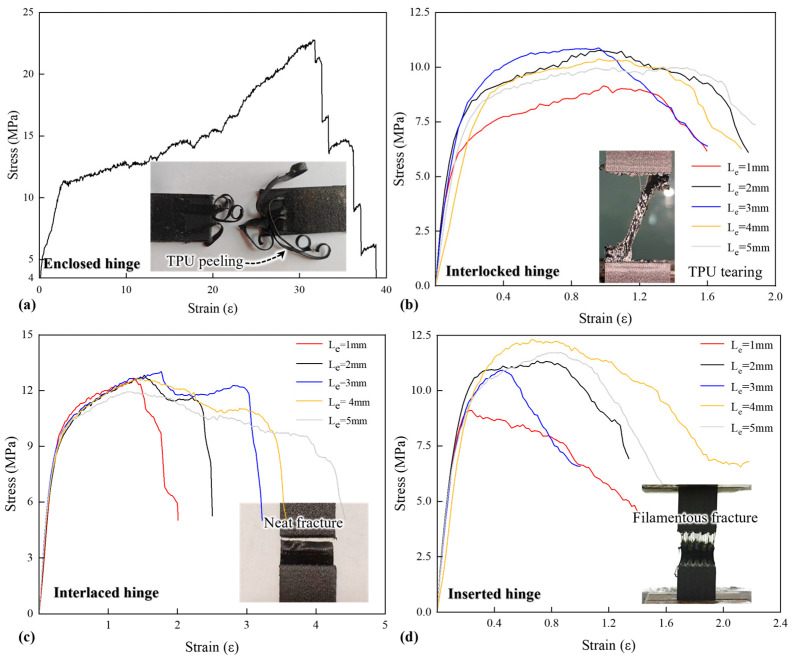
Tensile behavior of dual-material hinges: (**a**) enclosed hinge, (**b**) interlocked hinge, (**c**) interlaced hinge, (**d**) inserted hinge.

*Enclosed hinge*. As shown in Figure 6a, although the fully enclosed architecture reaches the highest ultimate tensile stress at 22.75 MPa, its usefulness for folding is clearly limited by its failure process. Under tension, the structure shows a distinct three-stage response. In the initial stage, interfacial delamination begins at 260% of elongation. Later on, the elastomeric phase carries the load independently. Eventually, the structure fails due to brittle fracture. This early peel-type delamination damages the integrity of the joint long before final failure occurs. In addition, the large volume of elastomer makes the hinge bulky in the out-of-plane direction. This bulkiness interferes with the precise folding motion required for deployable origami arrays.

*Interlocked hinge*. As shown in Figure 6b, the interlocked hinges show stress–strain curves that are typical of ductile polymer composites. The response includes three stages. The first stage is linear elastic elongation from 0 to 16%. The second stage is localized yielding from 0.8 to 16%. The third stage is an extended plastic flow plateau. The initial stiffness before yielding does not change with embedment depth. This result indicates that the initial modulus is controlled mainly by the intrinsic properties of the TPU core and PA6-CF boundaries, rather than by the interfacial geometry. However, the ultimate tensile capacity shows clear instability. The 1.0 mm group reaches an unusually low peak strength of 9.10 MPa. The specimens with intermediate embedment depths of 2.0–3.0 mm perform better than those with deeper embedments of 4.0–5.0 mm. This variation comes from mesostructural voids created by the complex multidirectional FDM toolpaths. Although the joints finally fail through diagonal rupture of the TPU rather than large-scale sliding, these printing defects act as strong stress concentrators.

*Interlaced hinge*. As shown in Figure 6c, in contrast, the interlaced cross-laminar architecture shows very stable load-bearing behavior. The peak tensile stress remains nearly constant at about 12.5 MPa across all tested embedment depths from 1.0 to 5.0 mm. The main mechanical advantage of this design lies in its high energy dissipation capacity. Increasing the embedment depth does not raise the peak strength. Instead, it greatly delays final fracture and significantly improves structural toughness. The fracture elongation increases steadily from 100.8% at a 1.0 mm embedment depth to 342.4% at a 5.0 mm depth. The longer embedment acts as a distributed energy-absorbing region. This region allows stress to transfer more gradually from the stiff PA6-CF to the compliant TPU. As a result, it reduces localized stress concentrations. This improved energy dissipation also changes the failure mode. Specimens with shallow embedments of 4.0 mm or less fail through interfacial shear and TPU pullout. By contrast, the specimen with the optimal 5.0 mm embedment fails through cohesive fracture within the TPU itself. This failure is marked by severe plastic necking and ductile tearing. These results confirm that the 5.0 mm interlaced geometry allows the TPU to reach its intrinsic mechanical limit before interfacial delamination begins.

*Inserted hinge*. As shown in Figure 6d, the inserted micro-rib architecture also fails to overcome defect-related limitations. Even though the FDM toolpaths are aligned in the longitudinal direction, the ultimate tensile strength remains highly nonlinear and inconsistent across different embedment depths. Fractographic observations show a mixed-mode failure process. The joints fail through both interfacial slippage and rupture of the elastomer. Increasing the embedment depth from 1.0 mm to 2.0 mm improves the ultimate strength by 28% in the thicker 1.8 mm specimens. However, the overall mechanical reliability of this design remains lower than that of the interlaced architecture.

**Figure 7:**
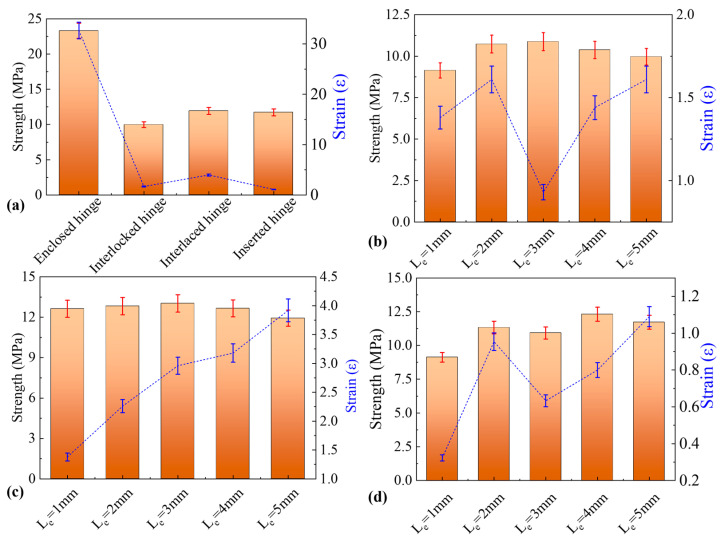
Comparison of the tensile performance of dual-material hinges: (**a**) overview of four distinct hinge hinges; (**b**) interlocked hinge, (**c**) interlaced hinge, (**d**) inserted hinge.

A comparative analysis of the four dual-material designs (Figure 7) identifies the interlaced architecture as the most effective kinematic element for multi-material origami. The interlocked and inserted designs show premature failure because toolpath complexity introduces void defects. The enclosed design also presents clear limitations. It adds excess material and fails early through peeling, which conflicts with lightweight design principles. By contrast, the interlaced hinge overcomes these major bottlenecks. This design uses continuous cross-laminar deposition to reduce intra-layer defects. The 5.0 mm interlaced configuration also improves structural toughness through cohesive energy dissipation. At the same time, it maintains the strict dimensional accuracy required for deployable structures. For these reasons, we select the interlaced design as the base joint configuration for subsequent Miura-ori structure.

### 3.2. Compression Behavior of Miura-Ori Structures

**Figure 8:**
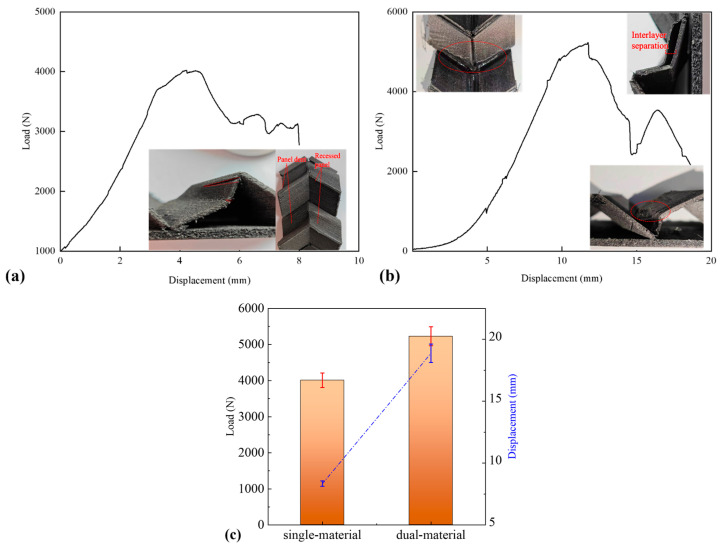
Compression behavior of Miura-ori structures: (**a**) single-material printed specimen, (**b**) dual-material printed specimen, (**c**) performance comparison.

Under compressive loading, the single-material printed Miura-ori structure shows a typical multistage mechanical response (Figure 8a). The response begins with a linear elastic stage. It then enters post-peak buckling, followed by an extended plateau and final densification. The structure reaches a peak compressive strength of 4019 N. The overall failure is controlled mainly by severe deformation of the panels. The panels develop an S-shaped buckling mode during loading. This deformation eventually causes catastrophic inter-layer delamination in the lower third of the span and macroscopic fracture near the upper boundary. This failure pattern reveals a basic limitation of out-of-plane FDM fabrication. The compressive strength of the structure is strongly restricted by the weak inter-layer bonding of the carbon-fiber composite. The structure also shows a globally symmetric failure shape. This pattern includes alternating outward bulging and inward collapse. The deformation reflects the inherent mountain-valley kinematics of the Miura-ori pattern [31]. The rigid fixed boundaries further intensify this geometric response.

By contrast, the Miura-ori structure with the optimized dual-material interlaced hinges shows a very different load-bearing mechanism and much better mechanical performance (Figure 8b). In this design, the rigid PA6-CF panels are fabricated by planar deposition instead of inclined spatial printing. As a result, the main compressive stress acts parallel to the longitudinal direction of the deposited filaments. This arrangement reduces the effect of weak z-axis inter-layer bonding, which is a common limitation in single-material printed structures. Because of this improvement, the ultimate compressive capacity increases to 5163 N, which is 28.5% higher than that of the single-material design. Although failure still occurs mainly in the lower third of the span, the failure mode changes clearly. The single-material structure fails through inter-layer delamination. In contrast, the dual-material structure fails through brittle rupture within the filaments. The dual-material architecture also shows greater deformability. It reaches an ultimate displacement of 18.7 mm, while the single-material configuration reaches only 8.0 mm. This result represents an improvement by a factor of 2.3.

As shown in Figure 8c, although the single- and dual-material Miura-ori structures show similar ultimate compressive strengths (4019 N vs. 5163 N, 28.5% increase), the dual-material structure displays much greater global deformability (8.0 mm vs. 18.7 mm, 133.8% increase). This improvement comes from the flexible hinges. These hinges make full use of the intrinsic hyperelasticity and ductility of the TPU phase. As a result, the dual-material structure converts local compliance into greater overall structural toughness. Under compressive loading, the hinged assembly can therefore undergo much larger geometric deformation before failure. It can also absorb much more energy prior to catastrophic collapse [32].

## 4. Discussion

The global folding behavior and load-bearing capacity of 3D-printed multi-material origami depend strongly on the local failure mechanics of the TPU–PA6-CF interface and the constituent materials. When the interfacial shear strength is low, as in shallow interlaced designs or fully enclosed architectures, damage is mainly controlled by peel-type delamination and clean pullout of the elastomer. In contrast, strong interfacial anchorage can shift the failure mode away from the interface. In this study, the optimized 5.0 mm interlaced embedment achieved that transition and forces failure into the bulk TPU matrix. Under these conditions, the elastomer shows a typical ductile fracture response. The material develops clear necking and cohesive tearing before final rupture. However, the ultimate fracture strain is often reduced by mesostructural defects created during FDM printing [33]. Internal voids and gaps between filaments act as strong stress concentrators. These defects accelerate crack growth and cause failure before the material reaches its theoretical elongation limit.

Within the rigid PA6-CF panels, the compressive failure pattern is governed by FDM-induced mechanical anisotropy and the overall geometry of the structure. In single-material printed Miura-ori structures, the panels are deposited in an inclined orientation. This printing path creates oblique weak planes in the material. Under compressive shear, these weak planes fail quickly through interlaminar separation. In contrast, the dual-material architecture uses planar-printed panels. This layout aligns the main compressive stress with the raster direction and the longitudinal axis of the carbon fibers. This alignment reduces the effect of out-of-plane interlaminar weakness. As a result, failure is delayed until the composite reaches its brittle limit, which is marked by fiber pullout and matrix cracking. The thin-walled panels also have high aspect ratios. This geometry makes them prone to macroscopic out-of-plane buckling. Even so, the dual-material structure performs better overall because of the hyperelastic behavior of the TPU phase. The dual-material architecture shows a slightly higher compressive capacity, with an increase of 28.5%. It also shows a much greater deformation capability, with an increase of 133.8% compared with the single-material printed structure. This synergy between material selection and structural design improves the global energy dissipation capacity of the origami system.

The macroscopic collapse of the Miura-ori structure is ascribed to the complex spatiotemporal development of local failure [34,35], especially for the stress concentration [36,37] at the discontinuous interface between the compliant TPU hinges and the rigid PA6-CF panels. Such failure usually occurs near the edge of the composite panel. This is because the interlaced geometry reduces the effective load-bearing cross section in the translon zone. The folding pattern of origami structure also plays an important role. Each tessellation defines its own kinematic path for deformation and stress transfer. In the Miura-ori pattern, the stress field follows a centrally symmetric distribution [8,38], indicating that the global folding geometry strongly controls where and how local material failure occurs.

## 5. Conclusions

This study investigated the integration of multi-material 3D printing and origami engineering, with a focus on rigid–flexible interfaces and the structural performance of dual-material Miura-ori architectures. The study addressed the key challenge of interfacial bonding between dissimilar material phases. Through this effort, the work establishes a practical framework for developing high-performance deployable structures with improved load-bearing capacity and energy dissipation. The main findings are summarized as follows.

(1) Comparative micromechanical testing identified the 5.0 mm interlaced, or cross-laminar, hinge as the most effective kinematic element for multi-material origami. This design overcomes the main problem of premature interfacial debonding. The extended interpenetrating interface distributes stress more evenly across the joint. As a result, the dominant failure mode shifts from weak interfacial separation to ductile fracture within the elastomer itself. This geometric optimization greatly improves system toughness. The fracture elongation increases from 100.8% at a 1.0 mm embedment depth to 342.4% at a 5.0 mm embedment depth. At the same time, the peak tensile strength remains stable at about 12.5 MPa.
(2) At the structural scale, the dual-material Miura-ori architecture shows much better performance than the single-material printed spatial structure. In the dual-material design, the main compressive stress is aligned with the longitudinal raster direction. This alignment reduces the out-of-plane interlaminar weakness that is common in spatial printing. As a result, the dual-material structure (5163 N, 18.7 mm) reaches a 28.5% higher compressive capacity and 133.8% higher fracture elongation compared with the single-material structure (4019 N, 8.0 mm).
(3) The optimized elastomeric hinges improve more than just peak load-bearing performance. These hinges allow the structure to make full use of the intrinsic hyperelasticity of the TPU phase. As a result, the dual-material system achieves much greater deformability and energy dissipation before final failure. These findings suggest that moving from single-material printing to optimized dual-material one is important for improving both the mechanical resilience and the functional adaptability of 3D-printed origami systems.

This study established coordinated control over dual-material FRP Miura-ori structures. The current FDM process remains subject to several inherent limitations. In particular, mesostructural defects (e.g., inter-filament voids) are readily introduced during fabrication, and localized tearing may occur at the interfaces between rigid and compliant phases. These factors constrain the attainable mechanical performance of the printed structures [39,40,41]. To address this issue, further improvement will require more advanced toolpath design strategies, potentially supported by machine learning techniques to minimize defect formation and improve interfacial reliability. Despite these limitations, this study offers meaningful insights for both fundamental research and practical engineering applications.

## Data Availability

The original contributions presented in this study are included in the article. Further inquiries can be directed to the corresponding authors.
